# Low-Cost Polyethylene Terephthalate Fluidic Sensor for Ultrahigh Accuracy Measurement of Liquid Concentration Variation

**DOI:** 10.3390/s21217410

**Published:** 2021-11-08

**Authors:** Ruey-Ching Twu, Kai-Hsuan Li, Bo-Lin Lin

**Affiliations:** Department of Electro-Optical Engineering, Southern Taiwan University of Science and Technology, Tainan 71005, Taiwan; firefist0108@gmail.com (K.-H.L.); a124749321@gmail.com (B.-L.L.)

**Keywords:** low cost, polyethylene terephthalate, fluidic sensor, birefringence, phase measurement, polarization interferometer, heterodyne scheme

## Abstract

A low-cost polyethylene terephthalate fluidic sensor (PET-FS) is demonstrated for the concentration variation measurement on fluidic solutions. The PET-FS consisted of a triangular fluidic container attached with a birefringent PET thin layer. The PET-FS was injected with the test liquid solution that was placed in a common path polarization interferometer by utilizing a heterodyne scheme. The measured phase variation of probe light was used to obtain the information regarding the concentration change in the fluidic liquids. The sensor was experimentally tested using different concentrations of sodium chloride solution showing a sensitivity of 3.52 ×10^4^ deg./refractive index unit (RIU) and a detection resolution of 6.25 × 10^−6^ RIU. The estimated sensitivity and detection resolutions were 5.62 × 10^4^ (deg./RIU) and 6.94 × 10^−6^ RIU, respectively, for the hydrochloric acid. The relationship between the measured phase and the concentration is linear with an R-squared value reaching above 0.995.

## 1. Introduction

Over recent years, flexible substrates based on polymers have been widely used in various optoelectronic components such as touch sensors, solar cells, photonic sensors, flexible displays, and radio frequency components, etc. [1,2,3,4,5,6]. Using the roll-to-roll process, polydimethylsiloxane microfluidic manufacturing and nano-imprinting technology have also become major approaches to producing miniaturized and disposable sensing units [7]. Among the various polymers including poly (methyl methacrylate), cyclic olefin copolymer, polyethylene terephthalate (PET), and polyimide [8], PET, a semicrystalline material, has the advantages of good heat resistance, chemical resistance, high transmittance, birefringence, and low cost. Most of the research on PET-related topics has focused on material characterizations and flexible device applications [8,9,10,11,12]. The birefringent characteristics can be fabricated as a wave plate and anti-reflection film [11,12]. To the best of our knowledge, this is the first report on the birefringent PET fluidic sensors for the measurement of concentration variation in a liquid solution.

In semiconductor manufacturing, it is essential to measure the concentration of chemical solutions and real-time process control [13]. The application seems to appear in the confirmation of certain chemical liquid storage tanks before the chemical liquid enters the process line. Sometimes, the wrong concentration of chemical solutions may lead to poor conduction of the wafer. Currently, a prism-based refractometer [14] based on total internal reflection and a metallic sensing head [15] are two useful contact-type techniques for online measurement of the concentration variations. However, a non-contact technique has advantages of remote monitoring and non-contamination during liquid transportation. In a closed container, this method can reduce the sampling and pollution of online products. The characteristics of dielectric constant and density are dependent on the concentration changes of liquid. To be able to quantitatively analyze changes in concentration through a non-contact technique, electromagnetic and ultrasonic waves have been widely used for the probe signals [16,17]. In some studies, the radiofrequency wave coupling scheme was applied to measure the shift of resonance frequency as well as the concentration variation [16]. However, the above-mentioned methods are relatively incapable of achieving high precision requirements. In the visible wavelengths, the change in refractive index caused by the concentration variation can be used to deflect the incident light while passing through a transparent container filled with the test solutions. The deflected beams are detected by a position sensitivity device or a beam profiler [18,19]. Although the methods are simple, the resolution is relatively limited. In addition, the change in the refractive index of solutions can also be estimated by analyzing the interference image pattern in a Mach–Zehnder interferometer [20]. Generally, low-cost glass or transparent plastic containers with round or square shapes are used to place the liquid as a disposable unit, and then different optical measurement principles are used to detect the variation in concentrations.

In this study, we propose a fluidic container attached with the birefringent PET to measure a phase-delay variation between two orthogonal polarizations of a probe light in a heterodyne interferometer. The PET was attached to the hypotenuse of a triangular container. Herein, the triangular container can enhance the change in refracted angle during the exchange of different liquids. The evaluated results indicate that the resolution of refractive index measurement is comparable with the commercial contact-type refractometer. Through this process, the purpose of low-cost, non-contact, and high-precision measurement can be achieved at the same time.

## 2. Sensing Principles and Birefringent Fluidic Sensor

**Figure 1:**
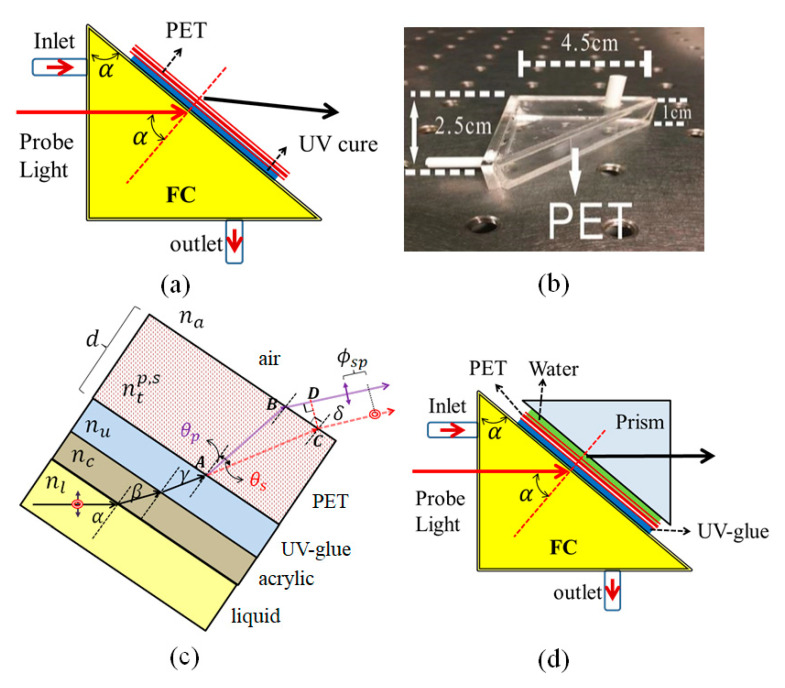
Schematic diagram of the sensing principle. (**a**) Birefringent polyethylene terephthalate (PET) fluidic sensor. (**b**) Photo of PET-FS. (**c**) The optical path for two orthogonal polarizations. (**d**) Prism coupling onto the FET-FS.

When the probe light with two orthogonal polarizations transmits through a birefringent material, the phase delay between them is dependent on the incident angle, birefringence, and the refractive index of the surrounding medium. Birefringence materials such as lithium niobate, titanyl phosphate, PANDA fiber, porous silicon, and liquid crystal have been successfully applied in the optical measurement fields including angle, displacement, refractive index, volatile organic, and biosensing diagnosis [21,22,23,24,25]. Usually, the birefringent sensors are placed in a common path polarization interferometer with the advantages of a simple and stable setup. Figure 1a explains the proposed birefringent PET fluidic sensor (PET-FS) and the probe light through the triangular fluidic container (FC) and UV curing attached PET layer. Figure 1b shows the image of PET-FS. The flowing liquid was injected through the inlet port and released to the outlet port driven by a peristaltic pump. The variation in thickness of PET was achieved by UV curing with a different number of single PET layers.

A schematic illustration of the transmitted light passing through the different mediums and the optical path is shown in Figure 1c. The probe light is normally incident on the front side of FC and the obliquely incident angle onto the hypotenuse of FC is denoted as *α*. After passing through the liquid in FC, the probe light transmits sequentially through the layered mediums including acrylic, UV-glue, and PET, then exits into the air. Between the PET and the air, it causes a total reflection at the incident angle α greater than a critical angle. To overcome the limitation of the incident angle, a prism coupling method with a thin layer of pure water is proposed as shown in Figure 1d. The coupled prism only enables a complete transmission of the probe light through the PET-FS. The probe phase variation is the same formula as without prism coupling. According to Snell’s law, the relations between refracted angle and each layer’s refractive index are presented as (Equations (1) and (2))

(1) $$n_{l}\cdot sin\alpha =n_{c}\cdot sin\beta =n_{u}\cdot sin\gamma =n_{t}^{s}\cdot sin\theta_{s}=n_{a}\cdot sin\delta$$

(2) $$n_{l}\cdot sin\alpha =n_{c}\cdot sin\beta =n_{u}\cdot sin\gamma =n_{t}^{p}\cdot sin\theta_{p}=n_{a}\cdot sin\delta$$

where the $n_{l}$, $n_{c}$, $n_{u}$, $n_{t}^{s,p}$, and $n_{a}$ represent the refractive indices of the test liquid, acrylic, UV-glue, PET, and air, respectively. Then, the refracted angle for the interface of acrylic and UV-glue is noted as β. The refracted angle for the interface of UV-glue and PET is noted as γ. The refracted angle for the interface of PET and air is noted as δ. In the isotropic mediums (liquid, acrylic, UV-glue, and air), both the input two polarizations (*p*-wave and *s*-wave) see the same refracted angles. In the birefringent PET layer, the different refracted angles $\theta_{s}$ and $\theta_{p}$ are represented for the *s*-wave and *p*-wave, respectively. In the case of $n_{t}^{s}<n_{t}^{p}$, $\theta_{s}$ is larger than $\theta_{p}$. The different refracted angles cause different paths in the PET and air mediums for two polarizations. To calculate the final phase difference between *s*-wave and *p*-wave, the phase values of s-wave and p-wave are defined with $\phi_{s}$ and $\phi_{p}$, as given in Equations (3) and (5), respectively, in which λ and d are the probe wavelength and thickness of the PET layer, respectively. The phase difference $\phi_{sp}$ is given as Equation (7). According to the formula, when the incident angle is fixed, the phase difference varies and the refractive index of liquid changes.

(3) $$\phi_{s}=\frac{2\pi }{\lambda }\cdot n_{t}^{s}\cdot \bar{AC}$$

(4) $$\bar{AC}=\frac{d}{cos\theta_{s}}$$

(5) $$\phi_{p}=\frac{2\pi }{\lambda }\cdot \left(n_{t}^{p}\cdot \bar{AB}+n_{a}\cdot \bar{BD}\right)$$

(6) $$\bar{AB}=\frac{d}{cos\theta_{p}};\;\bar{BD}=\bar{BC}\cdot sin\delta ;\;\bar{BC}=d\cdot \left(tan\theta_{s}-tan\theta_{p}\right)$$

(7) $$\phi_{sp}=\phi_{s}-\phi_{p}=\frac{2\pi }{\lambda }\cdot d\cdot \left(\sqrt{{\left(n_{t}^{s}\right)}^{2}-n_{l}^{2}\cdot sin^{2}\alpha }-\sqrt{{\left(n_{t}^{p}\right)}^{2}-n_{l}^{2}\cdot sin^{2}\alpha }\right)$$

Based on the proposed PET-FS, the refractive index measurement sensitivity (*RIMS*) is defined as differential operation for the curves of the phase variation versus RI, as expressed by

(8) $$RIMS=\left|d\phi_{sp}/dn_{l}\right|$$

Therefore, *RIMS* is mainly dependent on the incident angle, birefringence, and thickness of the PET layer. Additionally, the concentration measurement sensitivity (*CMS*) is defined as differential operation for the curves of the phase variation versus concentration ($c_{l}$), as expressed by

(9) $$CMS=\left|d\phi_{sp}/dc_{l}\right|$$

## 3. Measurement Setup

**Figure 2:**
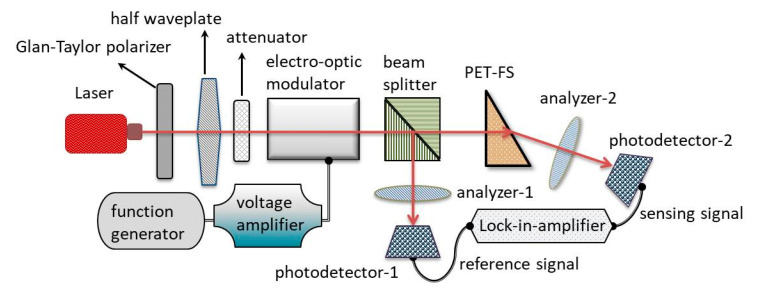
A setup for monitoring the fluidic concentration variations.

Figure 2 illustrates a schematic for the setup for monitoring the fluidic concentration variations based on a common path heterodyne interferometer [26]. The probe light is irradiated from a stabilized He–Ne laser. The intensity and linear polarizations can be adjusted by an attenuator, Glan–Taylor polarizer, and half waveplate. To improve the resolution of phase measurement, the phase-delay modulations between two orthogonal polarizations are generated after passing through an electro-optic modulator (EOM). The EOM is driven by a function generator and voltage amplifier. The amplitude and frequency of the applied sawtooth voltage are 150 V and 1 kHz. It can generate a similar sawtooth phase modulation. The modulated probe light is split into two paths in a beam splitter. The light power is 10 μW in both paths. The transmitted light, while passing through the PET-FS, is used as a sensing signal, and the reflected light is a reference signal. The liquids are injected into the PET-FS by a peristaltic pump. After the corresponding two analyzers, both interferometric signals are sinusoidal waves. They are received by the two photodetectors. Both the reference and the sensing signals are connected to a lock-in amplifier (SIGNAL RECOVERY: Model 7265). The lock-in amplifier also can be used as a phase meter, and the phase difference values between them can be obtained [26,27].

## 4. Results and Discussions

**Figure 3:**
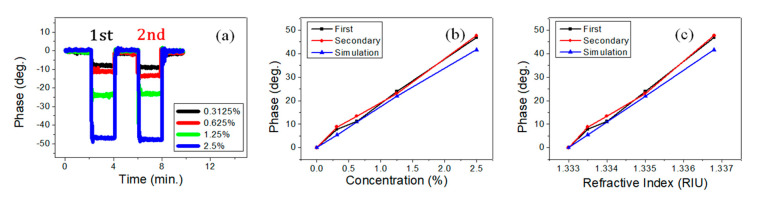
Measurements of NaCl solution concentrations with a single polyethylene terephthalate (PET) at 40° incident angle. (**a**) Phase variations by alternately exchanging the pure water and the different NaCl solution. (**b**) Phase versus NaCl concentration. (**c**) Phase versus the refractive index.

To evaluate the performance of the proposed PET-FS for measuring the variations in liquid concentrations, the test samples of sodium chloride (NaCl) solution with different concentrations were prepared by adjusting a weight ratio between NaCl and pure water. The flow rate was 120 SCCM (mL/min) driven by the peristaltic pump. In the first experiment, the used PET-FS was attached with a single PET of 188 μm thickness at an incident angle (α) of 40°. The PET was purchased from a local company (ruilong-glass.com). The RI values were measured by a commercial refractometer (A.KRÜSS: Model DR201-95; resolution: 10^−^^4^ RIU), and the variations in RI were in the range of 1.3328 to 1.3368 in NaCl concentrations ranging from 0% to 2.5%. The measured phase variations by alternately exchanging the pure water and the different NaCl solution are shown in Figure 3a. The mean phase values taken for 60 s in the middle period of each cycle were used to plot the figure of phase versus concentration, as shown in Figure 3b. The black line is the first cycle (1st). The red line is the secondary cycle (2nd). The blue line is the simulation result. The slope of the phase curve is defined as *CMS* (deg./%) through a linear fit. The average value of *CMS* was 18.47 deg./% for both cycles. The average value of R-squared was 0.996. Therefore, the phase measurement results show repeatability and good linearity. Figure 3c shows the phase versus RI for different concentrations. The slope of the phase curve is defined as *RIMS* (deg./RIU) through the linear fit. The average value of *RIMS* was 1.23 × 10^4^ deg./RIU for both cycles. The standard deviation of phase stability was 0.12 deg in the measurement system. The achievable measurement resolutions of concentration and RI were 9.8 × 10^−6^ RIU and 65 ppm, respectively.

**Figure 4:**
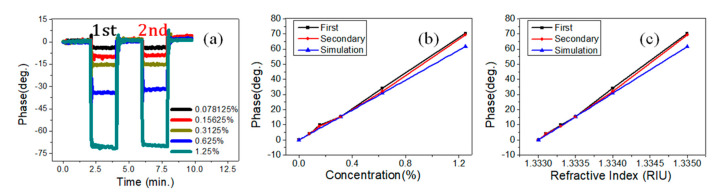
NaCl solution measurements with a single polyethylene terephthalate (PET) at 60° incident angle. (**a**) Phase variations by alternately exchanging the pure water and the different NaCl solution. (**b**) Phase versus NaCl concentration. (**c**) Phase versus the refractive index.

The measurement sensitivity can be enhanced by increasing the incident angle up to 60°, therefore, the experimental results obtained by adopting PET-FS coupling with a prism were as explained in Figure 1d. Between the FC and the coupling prism, thinly buffered pure water is present. Since the injected solutions are non-contact to the prism, and the water is easily removed by dry air. Therefore, the prism can be easily used for the next measurement. Figure 4a shows the phase variations by alternately exchanging the pure water and the NaCl solutions. The measured phase variation versus concentration is shown in Figure 4b. The black and red lines represent the first and secondary cycles, respectively. The blue line is the simulation result. The average value of *CMS* was 56.19 deg./% for both cycles. The average value of R-squared was 0.996. The phase measurement results showed repeatability and good linearity in the solution ranging from 0% to 1.25%. Figure 4c shows the phase variation versus RI for different concentrations of NaCl. The average value of *RIMS* was 1.23 × 10^4^ deg./RIU for both cycles. The standard deviation of phase stability was around 0.22 deg. The measurement sensitivity was 3.52 × 10^4^ (deg./RIU). The achievable resolutions of RI and concentration were 6.25 × 10^−6^ RIU and 39 ppm, respectively. The average value of R-squared was 0.998.

**Figure 5:**
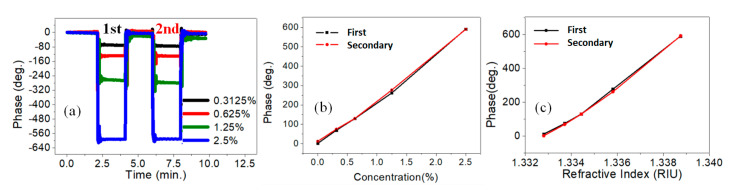
HCl solution measurements with five polyethylene terephthalate (PET) layers at 40° incident angle. (**a**) Phase variations by alternately exchanging the pure water and the different HCl concentration. (**b**) Phase versus concentration. (**c**) Phase versus the refractive index.

The measurement of NaCl solution can provide confident precision in the concentration variations. Generally, to measure acid-base corrosive liquids, a corrosion-resistant metallic head is used for the concentration measurement based on the conductivity measurement principle [15]. To further demonstrate the possibility of measuring the corrosive chemical liquids, the salt solution was replaced with different concentrations of hydrochloric acid (HCl). The used PET-FS was attached with five PET layers of 940 μm thickness and an incident angle of 40°. The thickness of the glue layer was around 150 μm between PET. The phase variations by alternately exchanging the pure water and the HCl solution are shown in Figure 5a. The mean phase values in the middle period of each cycle were used to plot the figure of phase versus concentration, as shown in Figure 5b. The black and red lines are represented for the first and secondary cycles, respectively. The average value of *CMS* was 233.14 (deg./%) after linear fit for both cycles. The average value of R-squared was 0.996. Figure 5c shows the phase versus RI for different concentrations. The average value of *RIMS* was 5.62 × 10^4^ (deg./RIU) for both cycles. In this measurement, the standard deviation of phase stability was around 0.39 deg. The achievable measurement resolutions of concentration and RI were 16.7 ppm and 6.94 × 10^−6^ RIU, respectively. In comparison with other published works, the resolution of the proposed sensor is better than the resolutions of $2.3\times 10^{-5}$ RIU ref. [18] and $3\times 10^{-4}$ RIU ref. [19].

The experimentations reveal that both a higher incident angle and increased PET thickness can improve the measurement sensitivity, but can also cause the deterioration in the system measurement stability, and limit the final resolution. Therefore, there must be an optimized choice of the incident angle and thickness of the PET layer.

## 5. Conclusions

Compared to the contact-type sensing head, the proposed non-contact measurement technique can reduce the post-experimentation cleaning step and avoid contaminating the sensing head. Furthermore, the PET-FS can be connected easily to the pipeline or storage container of chemical solutions for real-time monitoring of the liquid concentration variations. The dynamic ranges of measurement are tunable either by the thickness of the PET layer or the incident angle. According to the experimental results of this study, excellent merits such as being all-polymer, a compact size, and simple structure make the PET-FS promise high precision for chemical sensing applications. The use of acrylic injection molding technology makes it possible to produce various shapes of the high-transparency container attached with the PET layer. Compared with the generally disposable glass or acrylic containers used for the fluidic sample, the proposed PET-FS can use the same glass or acrylic containers added with one small piece of PET layer (1 cm × 2 cm) with UV-glue. The price of one PET with A4 size (29.7 cm × 21.0 cm) is around USD 20. The A4 size can be cut into at least 250 pieces (1 cm × 2 cm) so that the additional price for the small piece PET is around USD 0.08. The cost for the single PET-FS is still low. Moreover, the price is quite cheap under mass production.
